# Sentence retrieval for abstracts of randomized controlled trials

**DOI:** 10.1186/1472-6947-9-10

**Published:** 2009-02-10

**Authors:** Grace Y Chung

**Affiliations:** 1 Centre for Health Informatics, University of New South Wales, Sydney, NSW 2052, Australia

## Abstract

**Background:**

The practice of evidence-based medicine (EBM) requires clinicians to integrate their expertise with the latest scientific research. But this is becoming increasingly difficult with the growing numbers of published articles. There is a clear need for better tools to improve clinician's ability to search the primary literature. Randomized clinical trials (RCTs) are the most reliable source of evidence documenting the efficacy of treatment options. This paper describes the retrieval of key sentences from abstracts of RCTs as a step towards helping users find relevant facts about the experimental design of clinical studies.

**Method:**

Using Conditional Random Fields (CRFs), a popular and successful method for natural language processing problems, sentences referring to Intervention, Participants and Outcome Measures are automatically categorized. This is done by extending a previous approach for labeling sentences in an abstract for general categories associated with scientific argumentation or rhetorical roles: Aim, Method, Results and Conclusion. Methods are tested on several corpora of RCT abstracts. First structured abstracts with headings specifically indicating *Intervention*, *Participant *and *Outcome Measures *are used. Also a manually annotated corpus of structured and unstructured abstracts is prepared for testing a classifier that identifies sentences belonging to each category.

**Results:**

Using CRFs, sentences can be labeled for the four rhetorical roles with *F*-scores from 0.93–0.98. This outperforms the use of Support Vector Machines. Furthermore, sentences can be automatically labeled for *Intervention*, *Participant *and *Outcome Measures*, in unstructured and structured abstracts where the section headings do not specifically indicate these three topics. *F*-scores of up to 0.83 and 0.84 are obtained for *Intervention *and *Outcome Measure *sentences.

**Conclusion:**

Results indicate that some of the methodological elements of RCTs are identifiable at the sentence level in both structured and unstructured abstract reports. This is promising in that sentences labeled automatically could potentially form concise summaries, assist in information retrieval and finer-grained extraction.

## Background

The practice of evidence-based medicine (EBM) [1,2] asks clinicians to integrate clinical expertise with the best available external clinical evidence derived from scientific research, when making decisions about the care of individual patients. Reports of randomized controlled trials (RCTs) [3] are the primary evidence for treatment options, their efficacy, safety and possible adverse effects. But the number of reported RCTs has grown exponentially [4], and information retrieval in primary care is becoming more and more cumbersome. As a result, clinicians have large unmet information needs as they have little time to conduct searches and lack the right query formulation skills [5-7].

There is a clear need for better tools that could improve the precision of search results, thus increasing the likelihood for clinicians to find answers to clinical questions. At present, to aid clinicians access the best evidence, various manual efforts exist for summarizing findings derived from RCTs [8-11], and for encoding RCT protocols and outcomes into structured knowledge bases [12]. Ultimately these are labor intensive efforts for systematic reviewers, and can also benefit from better search engine design, and improved indexing.

Recognizing key sentences in scientific abstracts can be an important step for helping users to find relevant and important facts. Automatically extracted sentences can be used in information retrieval, they can be concatenated for automatically generated summaries or input to question-answering systems. Prior work by Ruch et al. [13] showed that key sentences in *Purpose *and *Conclusion *could be exploited to benefit information retrieval tasks.

The goal of this paper is to automatically locate key sentences about the methodology in RCT abstracts. By definition, RCTs compare treatment strategies on a clearly delineated population group, using a predefined set of outcome measures. When clinicians or reviewers are assessing the reliability of findings, RCT reports are scrutinized for sound design principles and reporting guidelines. Many journals now impose criteria for abstract structure for RCT reporting. The CONSORT statement [14] is a concerted effort to raise the quality of clinical trial reporting, through mandating a checklist of 22 items and a participant flow diagram in reports.

In this work, we hypothesize that RCT abstracts usually report the key methodological elements of *Intervention*, *Outcome Measure *and *Participants*, and we investigate whether these elements could be adequately identified at the sentence level using an automatic method. Some journals [15-17] already require the use of these specific section headings for RCTs to encourage clear documentation of key considerations. These usually break *Method *down into sub-components such as *Design*, *Setting*, *Interventions *and *Main Outcome Measures*. Yet most journals only require generic section headings of which these fall under the *Method *heading. Older RCT reports and indeed many current journals still allow unstructured abstracts. Thus the automatic categorization of *Method *sentences could be particularly useful for these cases.

The approach taken here models the natural ordering in discourse structure of a scientific abstract as a sequential machine, employing Conditional Random Fields (CRFs), a popular and successful method across a number of natural language processing (NLP) tasks. This paper will demonstrate that CRFs outperform a non-sequential approach, Support Vector Machines (SVM). Additionally, we will show that a previous effort to recognize the sequence of generic scientific argumentation (*Aim*, *Method*, *Results*, *Conclusion*) can be extended to locate the subtopics within the *Method *section in RCT reports.

The remainder of this paper is organized as follows. The next section outlines related approaches. This is followed by an elaboration of the method, including the data collection, sentence annotation, classification using CRFs and feature extraction. We will present a series of experiments that lead to the automated labeling of Intervention, Outcome Measures and Participants in both structured and unstructured abstracts, and subsequently discuss the results.

### Related Work

According to rhetorical structure theory [18], clauses in text relate to one another via relations such as Background, Elaboration, Contrast. These rhetorical relations when identified could be useful for information extraction, question answering, information retrieval and summarization. In NLP, researchers have attempted to recognize rhetorical relations using manually crafted and statistical techniques [19,20].

It has been claimed [21-23] that abstracts across scientific disciplines including the biomedical domain follow consistent rhetorical roles or "argumentative moves" (e.g. *Problem*, *Solution*, *Evaluation*, *Conclusion*). Teufel and Moens [24] has proposed a strategy for summarization by classifying sentences from scientific texts into seven rhetorical categories. Extracted sentences could be concatenated for automated user-tailored summaries.

Since then, several others have proposed to label sections of MEDLINE abstracts with four or five generic categories (*Background*, *Aim*, *Method*, *Results *and *Conclusion*), assigning structure to unstructured abstracts. Ruch et al. [25] used Naive Bayes to label sentences into the four main argumentative moves, with the goal of finding an appropriate *Conclusion *sentence which appears to be the most informative [26], and therefore best candidate to enhance search results. Other researchers have used Support Vector Machines (SVMs) [27-29], as well as Hidden Markov Models (HMMs) [30,31] which more effectively model the sequential ordering of sentences. Conditional random fields have been employed to recognize the four main rhetorical roles in our previous work [32] and also by Hirohata et al. [33].

Beyond the generic discourse level information, researchers have also investigated the extraction of key facts pertinent to clinical trials. In accordance with the PICO Framework [34], Patient, Intervention, Comparison and Outcome are the four dimensions that clinical questions can be reformulated to address. Demner-Fushman [35] has implemented an extractor for outcome sentences using an ensemble of classifiers, and Xu et al. [36] have reported the extraction of patient demographic information using a parser and HMMs.

In contrast to previous work, this paper explores the potential for identifying key sentences that are specific to RCT reports. In a study of medical journal abstracts, Dawes et al. [37] report that elements such as Patient-Population-Problem, Exposure-Intervention, Comparison, Outcome and Results were found in over 85% of the time. We seek to investigate here whether sentence categorization is sufficient for recognizing this information from both structured and unstructured abstracts. We specifically address sentences describing *Intervention*, *Participants *and *Outcome Measure*.

## Methods

### Data Collection

According to [3], an RCT may be defined as: "A prospective scientific experiment comparing the value of a treatment strategy in an experimental group with an alternative strategy in a control group, in which allocation to experimental or control group is determined by a chance mechanism."

To compile a data set of RCT abstracts, we rely on the publication type field in Pubmed. A broad search was conducted in MEDLINE for RCTs published between 1998 and 2006, specifying RCT in the publication type field. To obtain a representative cross-section of conditions the following keywords were used: asthma, diabetes, breast cancer, prostate cancer, erectile dysfunction, heart failure, cardiovascular, angina. Three data sets were prepared:

Set 1: A subset of RCTs was randomly selected to be manually annotated. These are both structured and unstructured. For the structured ones, the abstracts that contain headings that refer specifically to *Intervention*, *Outcome Measure *and *Participants *are removed from the set. Only abstracts with more general subheadings are included. As a result this test set contains 318 abstracts with 107 unstructured and 211 structured.

Set 2: A large data set of structured abstracts was collected. (13.6 k abstracts and 156 k sentences). All the section headings were mapped to one of four rhetorical roles (*Aim*, *Method*, *Results*, and *Conclusion*). Examples of original heading names are shown in Table 1.

**Table 1:** Headings of Structured Abstracts

Class	Example Heading Names
Aim	Goals, Objective, Purpose, Hypothesis, Introduction, Background, Context, Rationale

Intervention	Interventions, Interventions of the Study

Participants	Population, Patients, Subjects, Sample

Outcome Measures	Primary Outcome Parameters, Main Variables, Measures, Measurements, Assessments

Method	Materials, Study Design, Setting, Procedures, Process, Methodology, Research Design

Results	Results, Findings, Outcomes, Main Outcomes and Results

Conclusion	Conclusion, Conclusion and Clinical Relevance, Clinical Implications, Discussion, Interpretation

Examples of headings in structured abstracts that are mapped to equivalence classes for our classification purposes.

Sets I/O/P Three subsets were created from the main set (Set 2) of structured abstracts. All abstracts that contain a section heading referring to *Intervention *were compiled together into a data set, Set I. Each sentence in these abstracts was deterministically mapped to either one of the four rhetorical roles or the *Intervention *label. Other methodology related section headings such as *Setting*, *Design *etc were mapped to *Method*. Thus in this case *Method *labeled sentences are all the method related sentences excluding those that have been labeled as *Intervention*. In a similar manner, abstracts that contain a section heading referring to *Participants *were compiled together into a data set, Set P; abstracts containing a heading for *Outcome Measure *are compiled into Set O. Sets I/O/P are not mutually exclusive; some abstracts belong to all 3 sets. Abstracts with headings that combine more than one topic such as *Participants *and *Setting *are not included in these subsets. Examples of original heading names that map to each of the three categories are shown in Table 1. Set I contains 1575 abstracts, 21.2 k sentences; Set P contains 2280 abstracts, 29.8 k sentences; Set O contains 1740 abstracts and 22.9 k sentences.

### Sentence Annotation

Set 1 was manually annotated by the author. All sentences in the unstructured abstracts were labeled with one of the four generic rhetorical roles, *Aim*, *Method*, *Results *and *Conclusion*. For both the structured and unstructured abstracts, three additional types of sentences are annotated:

• **Intervention sentences**: In the abstracts, the allocation of a primary intervention and a control/placebo or secondary intervention are usually described, along with certain details of the protocol such as any blinding used, the dosage for drug interventions, frequency of administration, and duration of therapy. For non-drug therapies such as surgical and behavioral therapies and other multi-modal therapies, the method of administration and schedules of delivery are also specified.

All sentences referring to the assignment or randomization to treatments at each intervention arm, the method of administration, route of administration and other details of the protocol are labeled as *Intervention *sentences.

• **Outcome Measure sentences**: The efficacy and safety of an intervention are measured with outcome measures that should be clearly defined in the trial protocol. Also known as outcome assessments or endpoints, these consist of one or two primary measures and a set of secondary measures for consideration. All sentences that describe endpoints, methods for assessment and analysis techniques are labeled as such here.

• **Participant sentences**: RCTs are defined by strict eligibility criteria that require participants to have a specific clinical diagnoses, sex and/or age range. These inclusion/exclusion criteria and size of the recruited population are generally mentioned in the abstract.

Sentences that describe population size, clinical diagnoses, baseline characteristics are manually labeled as *Participant *sentences. Also labeled are sentences that mention the number of subjects enrolled, recruited, assigned and completed the trial.

In the structured abstracts, sentences in the above three categories are often found in the *Method *section. Each sentence can only be labeled with one of the four roles: *Aim*, *Method*, *Results *and *Conclusion*. But a sentence can simultaneously take on more than one of the labels: *Intervention*, *Participants *and *Outcome Measures*. For instance, the following sentence is labeled as both *Intervention *and *Outcome Measure*: "Patients received atorvastatin (10 mg daily) or placebo and were evaluated for cardiovascular and other outcomes over a median follow-up period of 3.9 years."

### Conditional Random Fields and Sentence Extraction as Sequence Labeling

Conditional random fields (CRFs) [38] are undirected graphical models. As discriminative models, CRFs describe the conditional distribution over a set of labels given the observed data. Formally, *X *is a random variable over the observation data, and *Y *is a random variable over the label set. Typically *X *and *Y *are concerned with sequential data where *X *= (*X*_1_, *X*_2_, ..., *X*_*n*_) are sequences of words or sentences, and *Y *= (*Y*_1_, *Y*_2_, ..., *Y*_*n*_) are sequences of labels such as part-of-speech tags. The CRF assigns the sequence of labels *y *to the observed input *X*. *X *and *Y *are jointly distributed, but CRFs directly model the conditional distribution *p*(*Y*|*X*) which takes the form:

(1) $$p(y|x)=\frac{1}{Z_{x}}exp\left(\sum_{j=1}^{N}\sum_{k=1}^{K}\lambda_{k}f_{k}(y_{j-1},y_{j},x,j)\right)$$

where *Z*_*x *_is the normalization factor, and *f*_*k *_is the feature function. Generally, it is assumed that the dependencies of *Y*, the state sequence, conditioned over *X *forms a linear chain.

CRFs are believed to offer several advantages over other sequence models such as HMMs: (1) as discriminative models, they do not model interdependence among observed data nor impose independence assumptions on the observations, (2) the framework lends itself to allow rich and unconstrained feature representations that could overlap or refer arbitrarily to the observations, and (3) better performance is obtained with CRFs as they are normalized over the full sequence, overcoming a well-known "label bias" problem [39]. In the past, CRFs have been applied to general NLP applications e.g. part-of-speech tagging [39], as well as biomedical text mining problems e.g. relation extraction [40], and named entity recognition [41].

In this paper, our implementation uses the Mallet package [42]. We use a Gaussian prior given in the default setting in Mallet. The problem of labeling with the four rhetorical roles is modeled as a first order linear chain of the four states, each one referring to Aim, Method, Results, Conclusion. For labeling with *Intervention*, *Participants *and *Outcome Measure*, a first order linear chain CRF is built for each problem. In each case, there are five states where one state represents the label in question, and the other four represent the four rhetorical roles. The feature vector for each state is derived from the observed sentence data and their syntactic features. The states will model the ordering of the sentences about Intervention, Participants and Outcome Measure in relation to Aim, other Method sentences, Results and Conclusion sentences.

We hypothesize that the CRF can better model the position of these sentences in the context of the four other rhetorical roles. For each case, the *Method *class/state would capture the sentences describing Method excluding those for the topic in question (Intervention, Participants or Outcome Measures.) To compare the performance of the sequence labeling, experiments are also conducted using a Support Vector Machine (SVM) classifier [43]. The SVM classifier uses SVMlight [44] with a linear kernel. SVMLight supports only binary classification, and a one-versus-all scheme is implemented to support *n*-ary classification.

### Features for Classification

#### Normalization

Prior to classification, each sentence undergoes normalization in which a script using regular expressions replaces complex numerical and mathematical notation into a canonical form or the semantic class. All integers and real numbers are mapped to symbols INT and REAL. All entities that represent measurements are normalized. For instance, a surface form of "200 mg/d" maps to MEASUREMENT. Ranges such as "200–300 mg/d" map to MEASUREMENT_RANGE. Statistical expressions such as *p*-values, confidence intervals, risk ratios, are mapped to a generic class STATISTICAL_EXPRESSION. Another common notation is population counts such as "*n *= 100" which has a semantic form POPULATION. Similarly, time and date and monetary expressions are also reduced to canonical form.

#### Word features

From previous work [32] and initial investigations, better performance was ascertained from using simple unigram bag-of-words, without further processing. Higher order *n*-gram features, stemming or removal of stop words did not improve performance.

#### Part-of-speech(POS) tags

For each sentence, a set of POS tags is derived from the output of the GENIA tagger [45], a POS tagger trained in the biomedical domain.

#### Positional Information

A normalized integer representing the sentence position from the beginning of the abstract is added to encode additional positional information.

#### Windowed features

The feature set from the previous sentence and the following sentence are included with the feature set of the current sentence. Features are marked accordingly with '-1' or '+1' to indicate previous or following sentence.

#### Rhetorical Roles

For the five class CRFs, it is possible to add the four rhetorical roles *Aim*, *Method*, *Results*, *Conclusion *as an additional feature. Each sentence in the abstract is given one of the four tags. In the structured abstracts, these are derived from the structured headings. In the unstructured abstracts, these are derived from the output of the four class CRF prediction.

### Experiments

#### Four Way Classification Experiments

Using the large Set 2, experiments are conducted to validate performance of CRFs on classifying four rhetorical roles. 15-fold cross-validation is performed on Set 2, comparing a baseline feature vector incorporating unigram bag-of-words, POS tags and positional information, with a windowed feature vector incorporating the features from the previous and following sentence. For comparison, results are also ascertained from the SVM classifier with the same two feature vectors.

#### Five Way Classification Experiments

Five way classification experiments are conducted on each of Sets O, I and P. 15-fold cross-validation is conducted in each case, comparing a baseline feature vector with one that incorporates the windowed features. Results are also ascertained for the SVM classifier with the same two feature vectors.

#### Four Way Classification on Manually Annotated Set

Using a CRF model trained on Set 2, performance is tested on the manually annotated test set, Set 1, for four way classification into the rhetorical roles.

#### Five Way Classification on Manually Annotated Set

Separate five way classification experiments are undertaken for each of the three cases with *Intervention*, *Outcome Measure *and *Participants*. Results are reported for four system configurations. These are described below:

S1: Baseline system where the feature vector incorporates windowed features.

S2: Baseline feature vectors are augmented with one of four rhetorical roles. These are derived from deterministic mappings if the abstract is structured. For unstructured abstracts, these labels are the predictions from the four way classifier.

S3: Baseline feature vectors are augmented with one of four rhetorical roles where for the unstructured abstracts, these are the manual labels from human annotations.

S4: Training data for each experiment are augmented with one of the sets: Set I, O or P.

For the Systems 1–3, 15-fold cross-validation is conducted. For System 4, during each test run, the training folds are augmented with the additional training data as described, and testing is conducted on each of the 15 folds. Results are reported for the entire set as well as the structured subset and unstructured subset separately.

## Results and discussion

### Results

For each experiment, we report, for each classification label, the precision, recall and the *F*-score. The *F*-score is computed as follows:

$$\begin{array}{c} P=\frac{\Sigma (TP)}{\Sigma (TP+FP)}\\ R=\frac{\Sigma (TP)}{\Sigma (TP+FN)}\\ F=\frac{2PR}{P+R} \end{array}$$

where *P *represents precision, *R *represents recall, *TP *is a true positive, *TN *is true negative, and *FP *is false negative.

We also report the accuracy, defined as the percentage of correctly labeled sentences for each data set in each experiment.

#### Four Way Classification Results

The four way classification on structured abstracts using 15-fold cross-validation achieve an accuracy of 93.53% for the baseline CRF system and a further 94.23% for the system with windowed data, as seen in Table 2. Windowed feature vectors clearly offer more information by incorporating lexical and syntactic contexts from the sentence before and after the current sentence. For the four classes corresponding to the rhetorical roles, *F*-scores range from 0.93 to 0.98 in the best system.

**Table 2:** Four Way Cross-Validation Sentence Classification Results on Structured Abstracts: Using CRFs

	With no windowed features			With windowed features		
	Accuracy = 93.53%			Accuracy = 94.23%		
	Precision	Recall	*F*-score	Precision	Recall	*F*-score

Aim	0.97	0.97	0.97	0.98	0.98	0.98
Method	0.93	0.92	0.92	0.94	0.93	0.93
Results	0.92	0.93	0.92	0.93	0.94	0.93
Conclusion	0.95	0.95	0.95	0.96	0.95	0.95

Sentence classification using CRFs into four major rhetorical roles. Results report using 15-fold cross validation for a system that uses no windowed features versus a system that uses windowed features. For this set, there were 13,610 abstracts, 156 k sentences.

In comparison with CRFs, SVMs under-perform, as seen in Table 3. Even when contexts are afforded by the windowed feature vectors, with positional information, SVMs do not model the sequential orderings as well as CRFs. The best SVM system achieved accuracy of 84.8% with *F*-scores ranging from 0.75 to 0.87.

**Table 3:** Four Way Cross-Validation Sentence Classification Results on Structured Abstracts: Using SVMs

	With no windowed features			With windowed features		
	Accuracy = 82.88%			Accuracy = 84.82%		
	Precision	Recall	*F*-score	Precision	Recall	*F*-score

Aim	0.84	0.77	0.80	0.86	0.80	0.83
Method	0.83	0.88	0.86	0.86	0.89	0.87
Results	0.85	0.87	0.86	0.85	0.89	0.87
Conclusion	0.76	0.68	0.72	0.79	0.72	0.75

Sentence classification using SVMs into four major rhetorical roles. Results report using 15-fold cross validation for a system that uses no windowed features versus a system that uses windowed

#### Five Way Classification Results

When evaluating with 15-fold cross-validation on five classes with the 3 subsets, Sets I/O/P, the same trends are exhibited as in the four-class results. Windowed features outperform features with no windows (Table 4), and CRFs outperform SVMs (Table 5).

**Table 4:** Five Way Cross-Validation Sentence Classification Results on Structured Abstracts: Using CRFs

	With no windowed features			With windowed features		
	Precision	Recall	*F*-score	Precision	Recall	*F*-score

1575 abs, 21.2 k sents	Accuracy = 86.30%			Accuracy = 87.99%		

Aim	0.99	0.99	0.99	1.00	0.99	1.00
Method	0.85	0.84	0.85	0.87	0.85	0.86
**Intervention**	**0.86**	**0.80**	**0.83**	**0.88**	**0.82**	**0.85**
Results	0.79	0.84	0.82	0.82	0.87	0.84
Conclusion	0.93	0.92	0.93	0.94	0.93	0.93

1740 abs, 22.9 k sents	Accuracy = 95.17%			Accuracy = 95.10%		

Aim	0.99	0.99	0.99	0.99	0.99	0.99
Method	0.97	0.97	0.97	0.96	0.97	0.97
**Outcome Measure**	**0.90**	**0.86**	**0.88**	**0.90**	**0.86**	**0.88**
Results	0.95	0.96	0.95	0.94	0.96	0.95
Conclusion	0.94	0.93	0.94	0.94	0.93	0.93

2280 abs, 29.8 k sents	Accuracy = 86.74%			Accuracy = 88.43%		

Aim	0.99	0.99	0.99	0.99	0.99	0.99
Method	0.85	0.86	0.85	0.87	0.87	0.87
**Participants**	**0.86**	**0.79**	**0.82**	**0.89**	**0.80**	**0.84**
Results	0.81	0.84	0.83	0.83	0.87	0.85
Conclusion	0.93	0.93	0.93	0.94	0.93	0.94

Sentence classification using CRFs into five classes, for each of the three classification problems. Results report using 15-fold cross validation for a system that uses no windowed features versus a system that uses windowed features.

**Table 5:** Five Way Cross-Validation Sentence Classification Results on Structured Abstracts: Using SVMs

	With no windowed features			With windowed features		
	Precision	Recall	-score	Precision	Recall	*F*-score

1575 abs, 21.2 k sents	Accuracy = 79.33%			Accuracy = 84.04%		

Aim	0.90	0.84	0.87	0.97	0.97	0.97
Method	0.80	0.82	0.81	0.84	0.82	0.83
**Intervention**	**0.79**	**0.72**	**0.75**	**0.83**	**0.78**	**0.80**
Results	0.75	0.80	0.77	0.78	0.83	0.80
Conclusion	0.79	0.74	0.77	0.88	0.87	0.87

1740 abs, 22.9 k sents	Accuracy = 85.14%			Accuracy = 91.12%		

Aim	0.90	0.83	0.86	0.96	0.95	0.96
Method	0.86	0.91	0.88	0.93	0.95	0.94
**Outcome Measure**	**0.81**	**0.80**	**0.80**	**0.85**	**0.81**	**0.83**
Results	0.86	0.88	0.87	0.91	0.92	0.92
Conclusion	0.79	0.72	0.75	0.87	0.87	0.87

2280 abs, 29.8 k sents	Accuracy = 80.64%			Accuracy = 85.20%		

Aim	0.89	0.82	0.86	0.96	0.95	0.95
Method	0.82	0.85	0.84	0.85	0.85	0.85
**Participants**	**0.80**	**0.72**	**0.76**	**0.87**	**0.76**	**0.81**
Results	0.76	0.81	0.79	0.79	0.84	0.82
Conclusion	0.82	0.85	0.84	0.88	0.87	0.87

Sentence classification using SVMs into five classes, for each of the three classification problems. Results report using 15-fold cross validation for a system that uses no windowed features versus a system that uses windowed features.

Using CRFs with windowed features, the best *F*-scores are 0.85 for Intervention sentences, 0.88 for Outcome Measure sentences and 0.84 for Participant sentences. The best *F*-scores for the other four classes in these three subsets range from 0.87 to 1.00. By comparison, using SVMs and windowed features, *F*-scores are 0.80 for Intervention sentences, 0.83 for Outcome Measure sentences and 0.81 for Participant sentences.

The actual identification of these types of sentences can be seen as a more confusable task than the four way problem because Intervention, Outcome Measure or Participant sentences could occur anywhere within the Method section, unlike the four way problem where the classes follow strictly the same ordering from Aim through to Conclusion. Nonetheless accuracies and *F*-scores are substantially higher when using CRFs compared with SVMs, reflecting that classification using sequence models is beneficial in recognizing the semantic classes of these sentences. A closer examination of the corpus shows that Outcome Measure sentences usually appears towards the end of the Method section following the mention of the intervention arms and participant characteristics. Intervention and Participants tend to be mentioned earlier within the Method section.

The variance in the ordering of these sentences stems from the differences in the subheading structures in structured abstracts of Sets I, O and P. The variance is due to the fact that many journals allow different heading structures and orderings.

#### Manually Annotated Set 1

In the manually annotated test set, there are 318 abstracts, 107 of which are unstructured. There are tagged 344 Intervention sentences, 341 Outcome Measure sentences, and 144 Participant sentences. In this corpus, 100% of the Outcome Measure sentences appear in the sentences also labeled as Method. 48 (33.3%) Participant sentences were identified in the sentences labeled as Results, and 29 Intervention sentences were identified in the sentences labeled also as Aim.

Clearly the frequency of occurrence of sentences that only discuss patient characteristics is lower. Baseline characteristics and inclusion criteria tend to be discussed in the Method section but the actual number recruited, participated and assessed are often reported in the Results section.

Examples of the sentences under the categories of Intervention, Outcome Measures and Participants in Set 1 are depicted in Table 6.

**Table 6:** Example Intervention, Outcome Measure and Participant Sentences

Intervention
Patients received either diltiazem, 240 mg/day, or amlodipine, 5 mg/day, for 2 weeks followed by diltiazem, 360 mg/day, or amlodipine, 10 mg/day, for 2 weeks. *Unstructured abstract*, *PMID: 11486240*

Participants were tested under two single-dose treatment conditions: placebo and citalopram (20 mg). *Unstructured abstract*, *PMID: 14731312*

Patients with node-positive (1–3) breast cancer were assigned to open-label epirubicin/vinorelbine (EV), epirubicin/vino-relbine and sequential paclitaxel (EV/T), epirubicin/cyclophosphamide (EC) or epiru-bicin/cyclophosphamide plus sequential paclitaxel (EC/T) therapy. *Method section*, *PMID: 14659328*

**Outcome Measure**

Standard treadmill exercise testing was the primary efficacy assessment. Patients also recorded incidence of angina attacks and use of glyceryl trinitrate spray. *Unstructured abstract*, *PMID: 11486240*

Arterial-coronary sinus differences of substrates were measured before cardiopulmonary bypass (CPB) and during early reperfusion. *Design section*, *PMID: 12775312*

The primary endpoints were overall survival (OS), relapse-free survival (RFS) and event-free survival (EFS). *Unstructured abstract*, *PMID: 12441265*

**Participants**

Twenty-eight healthy postmenopausal women, 16 without, and 12 with hormone replacement therapy (HRT) participated in this randomized, double-blind, cross-over study. *Unstructured abstract*, *PMID: 15994852*

Nineteen (19) young men, ages between 24 and 42, were enrolled in a single-center, institutional randomized, double-masked, crossover clinical trial. *Patients and Methods section*, *PMID: 15321024*

Twenty-four Chinese adults with type 2 diabetes participated. *Unstructured abstract*, *PMID: 15565080*

Examples of sentences labeled as Intervention, Outcome Measure and Participants in Set 1.

#### Four Way Classification on Manually Annotated Set

Results for four way classification on the manually annotated test set trained on the structured set, Set 2 are documented in Table 7. It is observed that *F*-scores range from 0.97 to 0.99 for the structured subset and 0.85 to 0.95 for the unstructured subset. In the unstructured subsets, the *F*-scores are 0.83 for Method and 0.85 for Aim. Thus, Method tends to be more difficult to recognize relative to the other classes.

**Table 7:** Four Way Sentence Classification Results on Manually Annotated Abstracts

	All Abstracts			Structured Subset			Unstructured Subset		
	Accuracy = 94.82%			Accuracy = 98.05%			Accuracy = 87.55%		
	P	R	*F*	P	R	*F*	P	R	*F*

Aim	0.98	0.91	0.94	0.99	0.99	0.99	0.96	0.78	0.85
Method	0.89	0.96	0.93	0.97	0.97	0.97	0.74	0.93	0.83
Results	0.97	0.94	0.96	0.98	0.98	0.98	0.95	0.85	0.90
Conclusion	0.97	0.99	0.98	0.99	0.99	0.99	0.94	0.97	0.95

Sentence classification using CRFs into four rhetorical roles on manually annotated data set. The CRF model was trained on 13.6 k set of structured abstracts. Precision (P), Recall (R) and *F*-score (*F*) are reported for each label over the entire data set (318), the structured subset (211) and unstructured subset (107).

#### Five Way Classification on Manually Annotated Set

Results for five way classification on the manually annotated set using CRFs and windowed feature vectors are depicted in Tables 8, 9 and 10, with each table representing results for each five way classification problem. Each table shows results for four different system configurations.

**Table 8:** Five Way Classification Including 'Intervention' on Manually Annotated Abstracts

	All Abstracts			Structured Subset			Unstructured Subset		
	
	P	R	*F*	P	R	*F*	P	R	*F*
**System 1**	Accuracy = 90.14%			Accuracy = 91.39%			Accuracy = 87.35%		
	
Aim	0.92	0.97	0.94	0.94	0.98	0.96	0.88	0.95	0.91
Method	0.85	0.81	0.83	0.86	0.83	0.84	0.80	0.76	0.78
**Intervention**	**0.87**	**0.78**	**0.82**	**0.88**	**0.80**	**0.84**	**0.85**	**0.74**	**0.79**
Results	0.91	0.97	0.92	0.91	0.95	0.93	0.89	0.92	0.90
Conclusion	0.96	0.94	0.95	0.98	0.94	0.96	0.92	0.94	0.93

**System 2**	Accuracy = 95.24%			Accuracy = 96.45%			Accuracy = 92.51%		
	
Aim	0.94	0.99	0.99	0.96	1.00	0.98	0.90	0.96	0.93
Method	0.92	0.91	0.92	0.93	0.93	0.93	0.89	0.87	0.88
**Intervention**	**0.87**	**0.79**	**0.83**	**0.88**	**0.80**	**0.84**	**0.85**	**0.75**	**0.80**
Results	0.98	0.99	0.99	0.99	1.00	0.99	0.96	0.97	0.96
Conclusion	0.99	0.99	0.99	1.00	1.00	1.00	0.89	0.87	0.88

**System 3**	Accuracy = 95.60%			Accuracy = 96.45%			Accuracy = 94.55%		
	
Aim	0.95	0.98	0.97	0.96	1.00	0.98	0.93	0.97	0.95
Method	0.92	0.92	0.92	0.93	0.93	0.93	0.91	0.91	0.91
**Intervention**	**0.87**	**0.80**	**0.83**	**0.88**	**0.80**	**0.84**	**0.86**	**0.78**	**0.82**
Results	0.98	0.99	0.99	0.99	1.00	0.99	0.97	0.99	0.98
Conclusion	0.99	0.99	0.99	1.00	1.00	1.00	0.99	0.98	0.99

**System 4**	Accuracy = 93.89%			Accuracy = 95.02%			Accuracy = 91.34%		
	
Aim	0.95	0.97	0.96	0.96	0.99	0.97	0.92	0.93	0.93
Method	0.88	0.89	0.88	0.89	0.90	0.90	0.85	0.85	0.85
**Intervention**	**0.77**	**0.71**	**0.74**	**0.78**	**0.73**	**0.75**	**0.76**	**0.69**	**0.72**
Results	0.99	0.99	0.99	1.00	1.00	1.00	0.96	0.96	0.97
Conclusion	0.99	0.99	0.99	1.00	1.00	1.00	0.96	0.98	0.97

Sentence classification using CRFs into five classes including *Intervention*. Results report on four systems. System 1: baseline system. System 2: feature vectors augmented with section headings from the four rhetorical roles, where they are either mapped from original headings in structured abstracts or predicted by the four class CRF model for unstructured abstracts. System 3 (oracle): feature vectors augmented with manually corrected section headings. System 4: same as System 2 except the training data is also augmented with training data from Set I. Precision (P), Recall (R) and *F*-score (*F*) are reported for each label over the entire data set (318), the structured subset (211) and unstructured subset (107).

**Table 9:** Five Way Classification Including 'Outcome Measure' on Manually Annotated Abstracts

	All Abstracts			Structured Subset			Unstructured Subset		
	
	P	R	*F*	P	R	*F*	P	R	*F*
**System 1**	Accuracy = 90.29%			Accuracy = 90.95%			Accuracy = 00.00%		
	
Aim	0.98	0.97	0.97	0.98	0.98	0.98	0.97	0.94	0.96
Method	0.87	0.83	0.85	0.88	0.83	0.85	0.85	0.82	0.84
**Outcome Measure**	**0.79**	**0.69**	**0.74**	**0.80**	**0.69**	**0.74**	**0.76**	**0.69**	**0.72**
Results	0.89	0.96	0.92	0.90	0.97	0.93	0.88	0.93	0.90
Conclusion	0.96	0.93	0.95	0.98	0.94	0.96	0.93	0.93	0.93

**System 2**	Accuracy = 94.22%			Accuracy = 94.89%			Accuracy = 92.70%		
	
Aim	0.98	0.98	0.98	0.98	0.99	0.99	0.96	0.95	0.96
Method	0.88	0.88	0.88	0.89	0.88	0.89	0.87	0.88	0.88
**Outcome Measure**	**0.81**	**0.77**	**0.79**	**0.82**	**0.77**	**0.79**	**0.80**	**0.75**	**0.77**
Results	0.97	0.98	0.98	0.98	0.99	0.99	0.95	0.97	0.96
Conclusion	0.99	0.99	0.99	1.00	1.00	1.00	0.97	0.97	0.97

**System 3**	Accuracy = 94.77%			Accuracy = 94.89%			Accuracy = 94.07%		
	
Aim	0.98	0.99	0.99	0.98	0.99	0.99	0.98	0.98	0.98
Method	0.89	0.89	0.89	0.89	0.88	0.89	0.90	0.88	0.89
**Outcome Measure**	**0.81**	**0.77**	**0.79**	**0.82**	**0.77**	**0.79**	**0.78**	**0.77**	**0.78**
Results	0.98	0.99	0.98	0.98	0.99	0.99	0.96	0.98	0.97
Conclusion	0.99	0.99	0.99	1.00	1.00	1.00	0.99	0.98	0.98

**System 4**	Accuracy = 95.60%			Accuracy = 96.71%			Accuracy = 93.09%		
	
Aim	0.99	0.98	0.98	1.00	1.00	1.00	0.96	0.95	0.96
Method	0.91	0.89	0.90	0.92	0.91	0.92	0.91	0.85	0.88
**Outcome Measure**	**0.82**	**0.85**	**0.84**	**0.85**	**0.86**	**0.85**	**0.76**	**0.83**	**0.79**
Results	0.99	0.99	0.99	1.00	1.00	1.00	0.96	0.97	0.96
Conclusion	0.99	0.99	0.99	1.00	1.00	1.00	0.97	0.98	0.97

Sentence classification using CRFs into five classes including *Outcome Measure*. Results report on four systems as described in Table 8. System 4 describes a system identical to System 2 except the training data is augmented with those from Set O.

**Table 10:** Five Way Classification Including 'Participants' on Manually Annotated Abstracts

	All Abstracts			Structured Subset			Unstructured Subset		
	
	P	R	*F*	P	R	*F*	P	R	*F*
**System 1**	Accuracy = 90.59%			Accuracy = 90.82%			Accuracy = 90.08%		
	
Aim	0.96	0.97	0.97	0.97	0.98	0.97	0.95	0.96	0.95
Method	0.86	0.91	0.88	0.87	0.92	0.89	0.83	0.89	0.86
**Participants**	**0.68**	**0.37**	**0.48**	**0.67**	**0.36**	**0.47**	**0.71**	**0.40**	**0.52**
Results	0.91	0.94	0.92	0.90	0.95	0.93	0.93	0.92	0.92
Conclusion	0.96	0.93	0.95	0.98	0.93	0.95	0.93	0.94	0.94

**System 2**	Accuracy = 94.64%			Accuracy = 94.98%			Accuracy = 93.68%		
	
Aim	0.98	0.98	0.98	0.99	0.99	0.99	0.97	0.97	0.97
Method	0.90	0.96	0.93	0.91	0.97	0.94	0.88	0.93	0.90
**Participants**	**0.76**	**0.40**	**0.52**	**0.76**	**0.37**	**0.50**	**0.77**	**0.46**	**0.58**
Results	0.96	0.98	0.97	0.96	0.99	0.98	0.96	0.96	0.96
Conclusion	0.99	0.99	0.99	1.00	1.00	1.00	0.97	0.97	0.97

**System 3**	Accuracy = 94.91%			Accuracy = 94.98%			Accuracy = 94.75%		
	
Aim	0.98	0.98	0.98	0.99	0.99	0.99	0.97	0.97	0.97
Method	0.90	0.96	0.93	0.90	0.97	0.93	0.88	0.94	0.91
**Participants**	**0.77**	**0.38**	**0.51**	**0.76**	**0.37**	**0.49**	**0.80**	**0.43**	**0.56**
Results	0.97	0.97	0.98	0.96	0.99	0.98	0.97	0.98	0.98
Conclusion	0.99	0.99	0.99	1.00	1.00	1.00	0.99	0.98	0.98

**System 4**	Accuracy = 94.25%			Accuracy = 94.98%			Accuracy = 94.75%		
	
Aim	0.99	0.99	0.99	1.00	1.00	1.00	0.97	0.96	0.97
Method	0.92	0.94	0.93	0.93	0.94	0.94	0.89	0.92	0.90
**Participants**	**0.56**	**0.38**	**0.45**	**0.54**	**0.34**	**0.42**	**0.59**	**0.51**	**0.55**
Results	0.96	0.98	0.97	0.95	1.00	0.97	0.97	0.95	0.96
Conclusion	0.99	0.99	0.99	1.00	1.00	1.00	0.95	0.98	0.97

Sentence classification using CRFs into five classes including *Participants*. Results report on four systems as described in Table 8. System 4 describes a system identical to System 2 except the training data is augmented with those from Set P.

For system 1, it is seen that *F*-scores are 0.82 for Intervention sentences, 0.74 for Outcome Measure sentences and 0.48 for Participant sentences. Clearly Participant sentences are more difficult to identify. Recall suffers considerably for these sentences.

System 2 adds information about section headings from the four rhetorical roles. This raises performance to 0.83 for Intervention, 0.79 for Outcome Measures and 0.52 for Participant sentences. In system 2, the four class labels given to the structured subset are deterministic mappings but the labels assigned to unstructured subsets are derived from the four class CRF tagger, which introduces some errors. However, improvements are seen for both structured and unstructured parts. This is particularly pronounced in recall for Outcome Measure (0.69 to 0.77) and in precision for Participants (0.68 to 0.76).

In System 3, the four rhetorical roles are assigned manually to the unstructured abstracts and added to the feature vectors, and hence this reflects an oracle system in which the added information from rhetorical roles is error free. This effected a small improvements in the *F*-scores for unstructured abstracts for Intervention and Outcome Measures. Since gains are quite small, it demonstrates that the automatic assignment of the rhetorical roles are sufficient to enable some gains in the tagging of the Intervention, Outcome Measure and Participant sentences.

In System 4, Sets I/O/P are respectively added to the training data for building the CRF models during cross-validation on the manually annotated test set, Set 1. This is intended to automatically increase the size of the training data by using data derived from structured abstracts which had sentences labeled with the respective headings of interest. The results are mixed as this degraded *F*-scores for Intervention from 0.83 (System 3) to 0.74 (System 4), and 0.52 (System 3) to 0.45 (System 4) for Participants. But for Outcome Measure sentences, the *F*-scores improved markedly from 0.79 (System 2) to 0.84 (System 4), and particularly for recall (0.77 to 0.85). This showed that the structured data from Set O is well matched to Set 1 for the Outcome Measure sentences but it is likely that the location of Intervention and Participant sentences differ between the structured sets and Set 1.

## Discussion

Our experiments in four way classification are competitive with previous results. Hirohata et al. [33] achieved up to 95.5% accuracy and *F*-scores from 0.94 to 0.98 on a similar task for their data set. Their data set of MEDLINE abstracts was not the same as ours and is therefore not directly comparable. The goal was to understand if sentence level extraction is possible for some of these key parameters, and we find that Intervention and Outcome Measure are both possible, and that performance improved for Outcome Measure when training data was augmented with structured abstracts containing Outcome Measure sentences. It can be inferred that at the sentence level Intervention and Outcome Measure sentences can be extracted.

However, the identification of Participant sentences achieved poorer performance. Further examination indicates that patient characteristics such as their diagnoses are often embedded throughout the abstract in the Intervention sentences. An example is: "Patients with node-positive (1–3) breast cancer were assigned to...." These sentences were not labeled as Participant sentences. As a result, there are fewer sentences that primarily describe patient characteristics. Alternative methods for identifying specific patient information such as population number, age, gender, condition is likely to yield better results. Unstructured abstracts are likely to vary more in writing styles, and thus performance suffers by a small amount in all cases. The rhetorical roles of such sentences are not as distinct since authors are not forced to write Aim and Method in separate sentences. Many unstructured abstracts have the tendency to compress the statement of objectives and the intervention treatment and measurements entirely into a single sentence. For example in Table 11, the first sentence encompasses the the statement of objectives as well as the description of the intervention. All following sentences present experimental findings and interpretation. There is some evidence that it is easier to identify Intervention, Outcome Measure and Participant sentences in structured abstracts, as seen in the five way classification results for Set 1 in Tables 8, 9 and 10. It can be inferred that imposing even some general structure in accordance to the four rhetorical roles can improve the ability for a machine to identify the three elements of Methodology in RCTs. It would seem that the sequential scheme is an advantage to the *n*-ary classification framework. We believe CRFs and a sequential model are a suitable framework for this problem as Intervention, Outcome Measure and Participant sentences may not always appear in the Method section. Our model accounts for the sequential ordering in the abstract in recognizing the four rhetorical roles along with the sentence topics together. This avoids making a hard decision on labeling the sentences with one of the four rhetorical roles and only looking for the method related sentences in the Method section.

**Table 11:** Example Unstructured Abstract

To determine the mechanisms underlying increased aerobic power in response to exercise training in octogenarians, we studied mildly frail elderly men and women randomly assigned to an exercise group (n = 22) who participated in a training program of 6 mo of physical therapy, strength training, and walking followed by 3 mo of more intense endurance exercise at 78% of peak heart rate or a control sedentary group (n = 24). Peak O2 consumption (V(O2 peak)) increased 14% in the exercise group (P 0.0001) but decreased slightly in controls. Training induced 14% increase (P = 0.027) in peak exercise cardiac output (Q), determined via acetylene re-breathing, and no change in arteriovenous O2 content difference. The increase in Q was mediated by increases in heart rate (P = 0.009) and probably stroke volume (P = 0.096). ... *PMID: 12857764*

### Limitations

One limitation for this work is that only one person (the author) was engaged in manually annotating the test set (Set 1) so that inter-annotator agreement cannot be obtained. Inter-annotator agreement would be useful in countering any inherent bias that is introduced from using just one annotator.

## Conclusion

This work has demonstrated that some elements of RCT methodology can be automatically identified in RCT abstracts at the sentence level. Using the sequential framework of Conditional Random Fields (CRFs), sentences in abstracts are labeled with the four rhetorical roles of *Aim*, *Method*, *Results *and *Conclusion*. CRFs are shown to outperform Support Vector Machines.

Promising performance was obtained in recognizing sentences that describe intervention arms (*F *= 0.83) and the primary and secondary outcome measures or endpoints (*F *= 0.84) in an human annotated set of both structured and unstructured abstracts.

## Competing interests

The author declares that they have no competing interests.

## Authors' contributions

The author GYC solely designed and implemented the system, performed data collection and annotation, carried out the experiments and analyses, and drafted the manuscript.

## Pre-publication history

The pre-publication history for this paper can be accessed here:

http://www.biomedcentral.com/1472-6947/9/10/prepub
